# Supplementary material for: Peptide-Functionalized Nanoemulsions as a Promising Tool for Isolation and Ex Vivo Culture of Circulating Tumor Cells
Source: Bioengineering (Basel). 2022 Aug 10;9(8):380. doi: 10.3390/bioengineering9080380 (PMC9405120; doi:10.3390/bioengineering9080380)
Supplement: Supplementary file 1 [file bioengineering-09-00380-s001.zip › bioengineering-1825224-supplementary-conversion.pdf]

bioengineering

MDPI

*Communication*

# Peptide-Functionalized Nanoemulsions as a Promising Tool for Isolation and Ex Vivo Culture of Circulating Tumor Cells

**Nuria Carmona-Ule [1], Noga Gal [2], Carmen Abuín Redondo [1,3], María De La Fuente Freire [4,5,6], Rafael López López [1,3,4,6]*and Ana Belén Dávila-Ibáñez [1,3,4]***

1 Roche-Chus Joint Unit, Translational Medical Oncology Group (Oncomet), Health Research Institute of Santiago de Compostela (IDIS), Hospital Gil Casares, 15706 Santiago de Compostela, Spain; nuria.carmona.ule@gmail.com (N.C.-U.); carmen.abuin.redondo@sergas.es (C.A.R.); rafael.lopez.lopez@sergas.es (R.L.L.)

2 Interdisciplinary Nanoscience Center (iNANO), Aarhus University, 8000 Aarhus, Denmark; noga@inano.au.dk

3 Translational Medical Oncology Group (Oncomet), Health Research Institute of Santiago de Compostela (IDIS), University Clinical Hospital of Santiago de Compostela (SERGAS), 15706 Santiago de Compostela, Spain

4 Cancer Network Research (CIBERONC), 28029 Madrid, Spain; maria.de.la.fuente.freire@sergas.es

5 Nano-Oncology Unit, Health Research Institute of Santiago de Compostela (IDIS), SERGAS, 15706 Santiago de Compostela, Spain

6 DIVERSA Technologies S.L., 15782 Santiago de Compostela, Spain

* Correspondence: ana.belen.davila.ibanez@sergas.es

**Supplementary Materials:** The following are available online at www.mdpi.com/xxx/s1, Figure S1: Analysis of final concentration in Pep10-NEs and GE11-NEs formulations, Figure S2: Analysis of the stability after storage for 3 months at 4 °C, particle size, PdI and ζ-Potential values for Pep10-NEs and GE11.

**A**

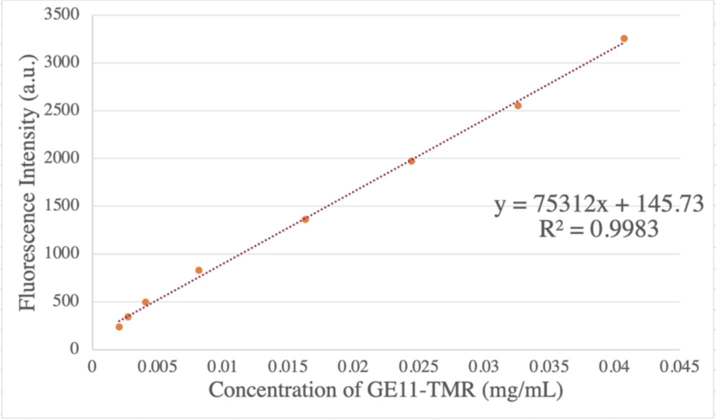


| GE11(TMR) in 1mL formulation | | | | |
|---|---|---|---|---|
| Concentration added GE11 (mg/mL) | | | | |
| 0.0163 | | | | |
| | n1 | n2 | n3 | n4 |
| Concentration1 | 0.005208599 | 0.003376222 | 0.003522281 | 0.004265854 |
| Concentration2 | 0.005248433 | 0.003442612 | 0.00373473 | 0.004199464 |
| Concentration3 | 0.004929759 | 0.003137216 | 0.003588671 | 0.003880789 |
| Average | 0.00512893 | 0.003318683 | 0.003615227 | 0.004115369 |
| | 0.004044552 | | | |

**B**

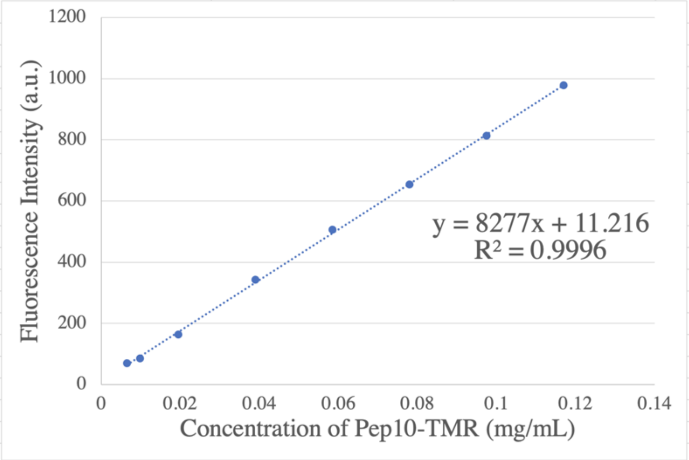


| Pep10(TMR) in 1mL formulation | | | | |
|---|---|---|---|---|
| Concentration added Pep10 (mg/mL) | | | | |
| 0.0195 | | | | |
| | n1 | n2 | n3 | n4 |
| Concentration1 | 0.008793524 | 0.009518425 | 0.009880875 | 0.009155974 |
| Concentration2 | 0.008914341 | 0.009035158 | 0.010243325 | 0.008914341 |
| Concentration3 | 0.009397608 | 0.009155974 | 0.009397608 | 0.009035158 |
| Average | 0.009035158 | 0.009236519 | 0.009840602 | 0.009035158 |
| | 0.009286859 | | | |

**Figure S1.** Analysis of final concentration in Pep10-NEs and GE11-NEs formulations.

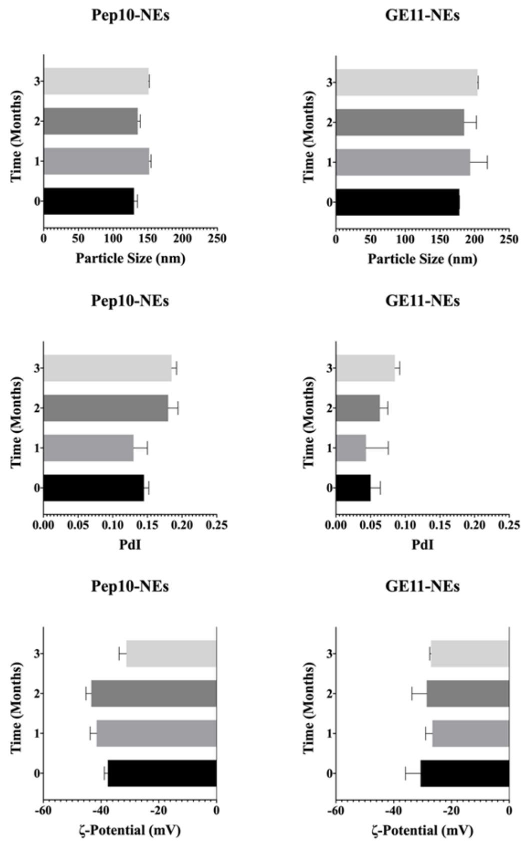


**Figure S2.** Analysis of the stability after storage for 3 months at 4 °C, particle size, PdI and ζ-Poten-tial values for Pep10-NEs and GE11.
